# Supplementary figures and images for: Monetary policy reaction function: A Bayesian analysis for the BRICS
Source: PLoS One. 2024 Aug 28;19(8):e0307436. doi: 10.1371/journal.pone.0307436 (PMC11355566; doi:10.1371/journal.pone.0307436)

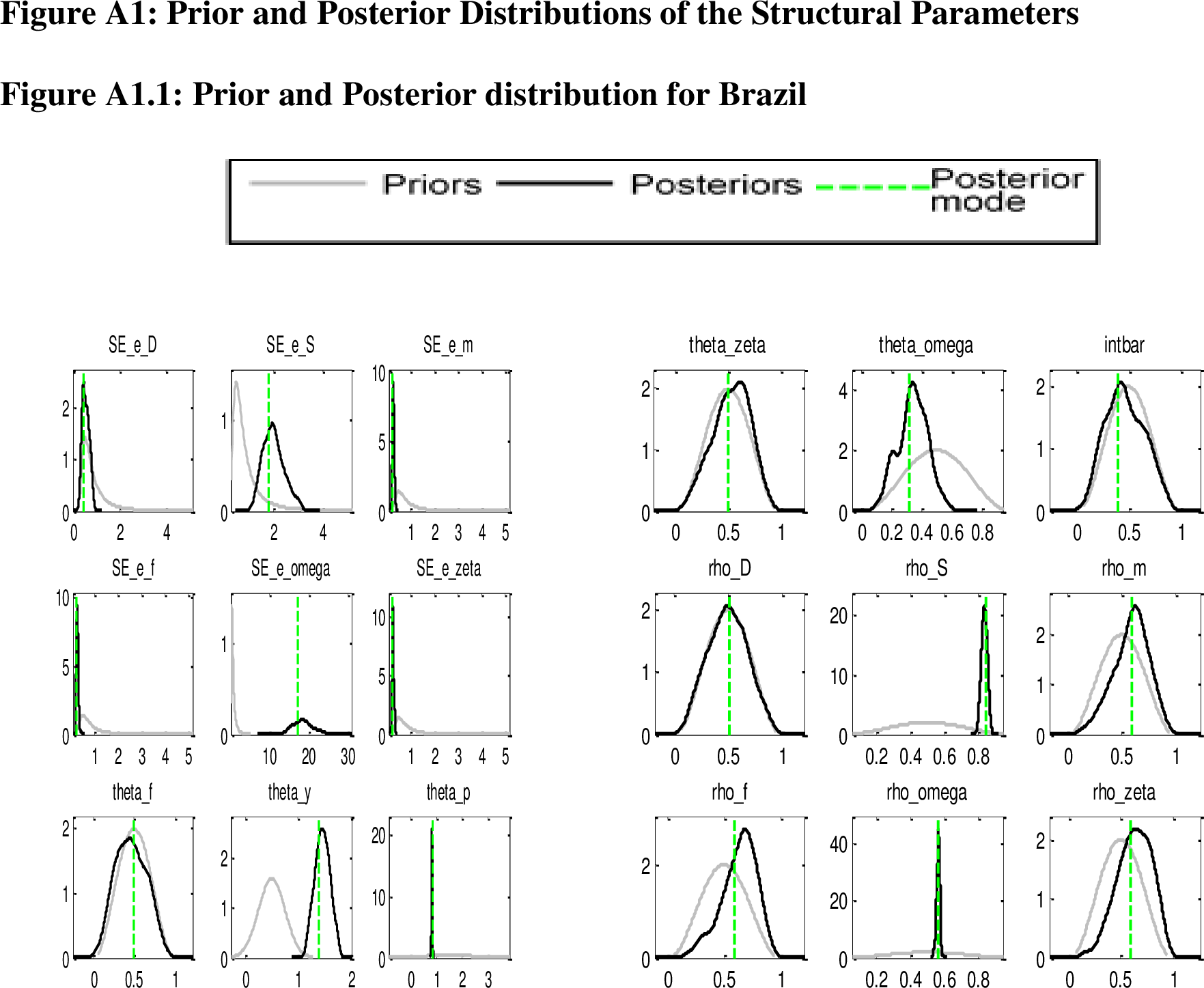

Supplement: S1 Fig — (ZIP) [file pone.0307436.s001.zip › Fig A1.1.tif]

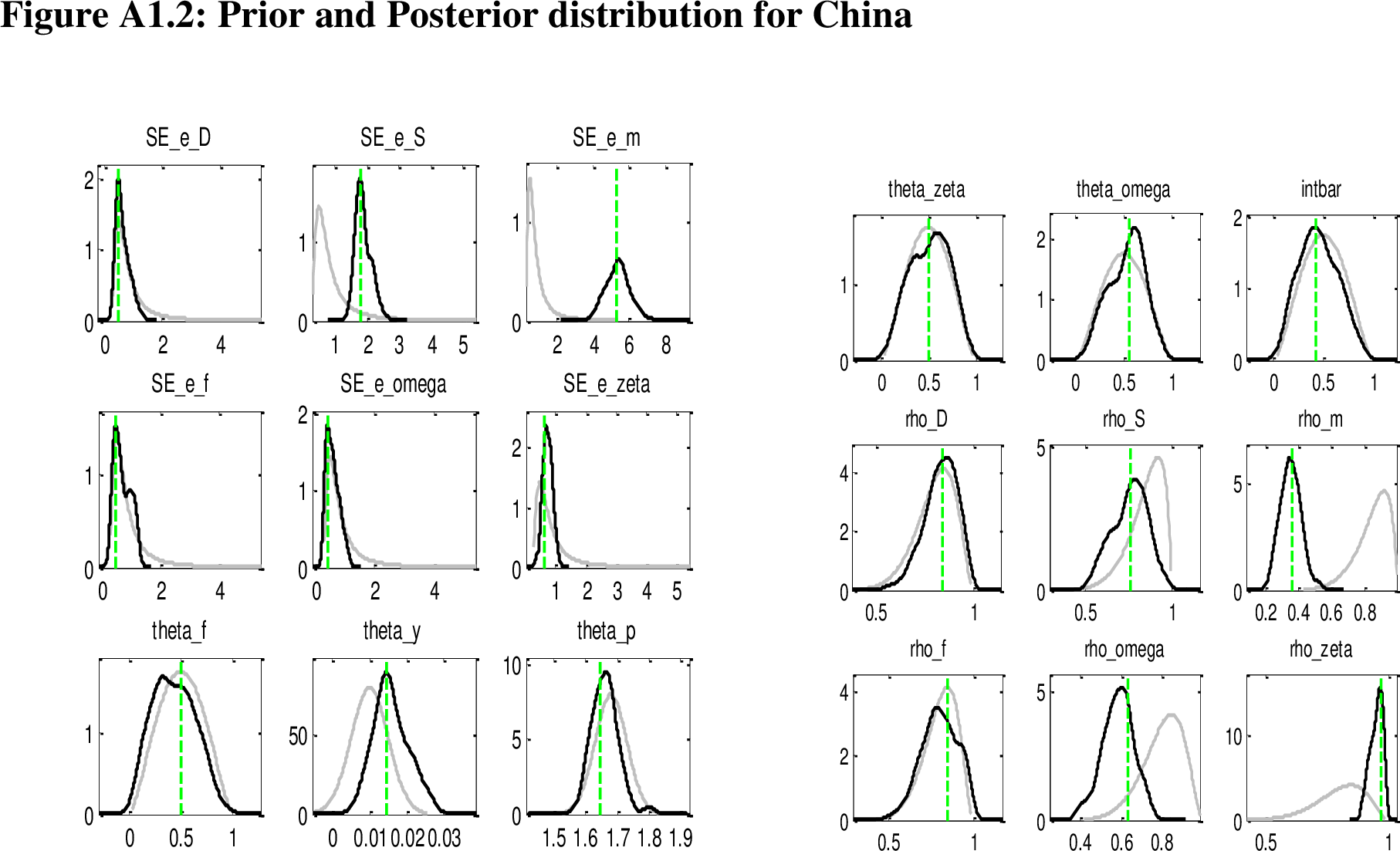

Supplement: S1 Fig — (ZIP) [file pone.0307436.s001.zip › Fig A1.2.tif]

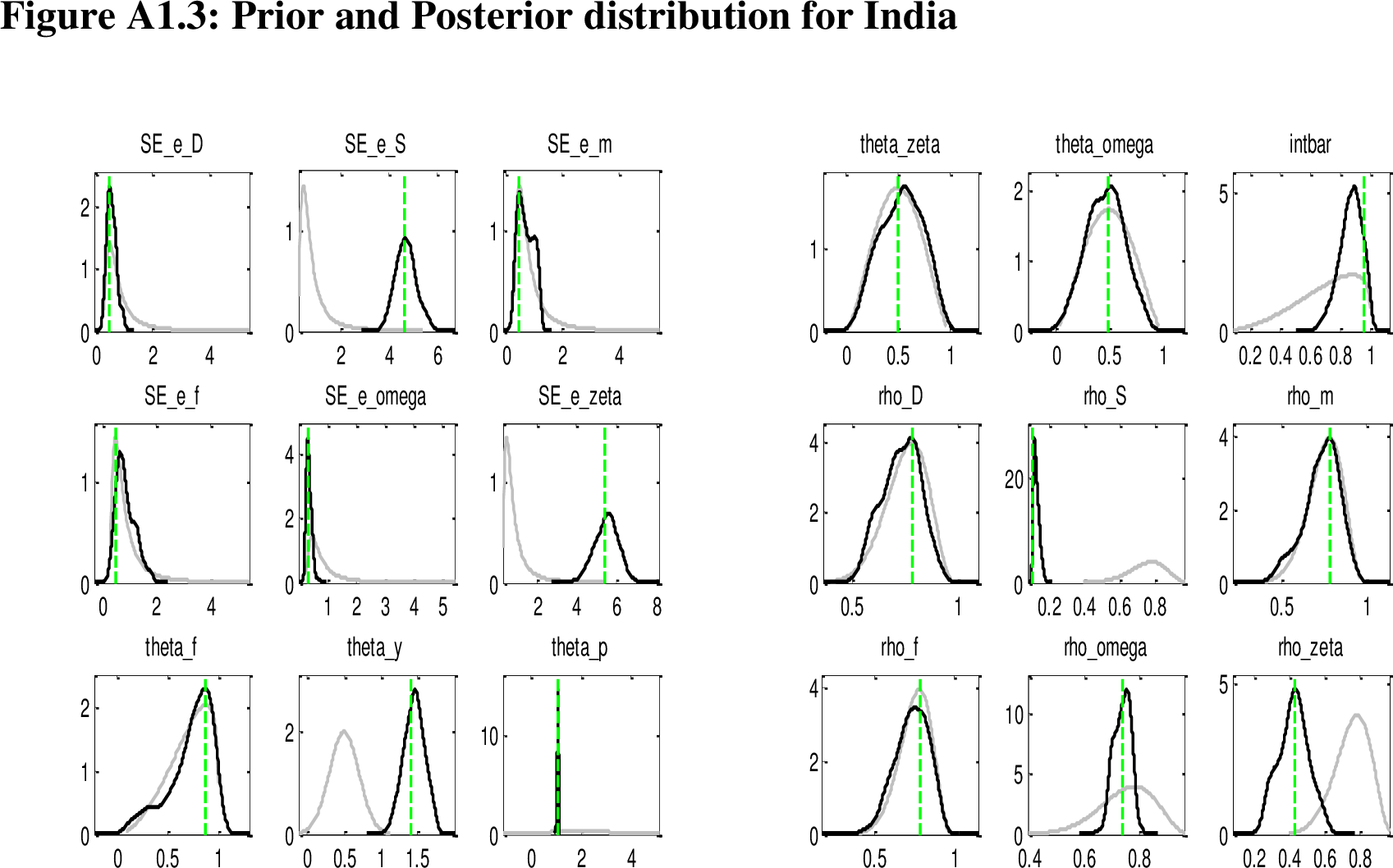

Supplement: S1 Fig — (ZIP) [file pone.0307436.s001.zip › Fig A1.3.tif]

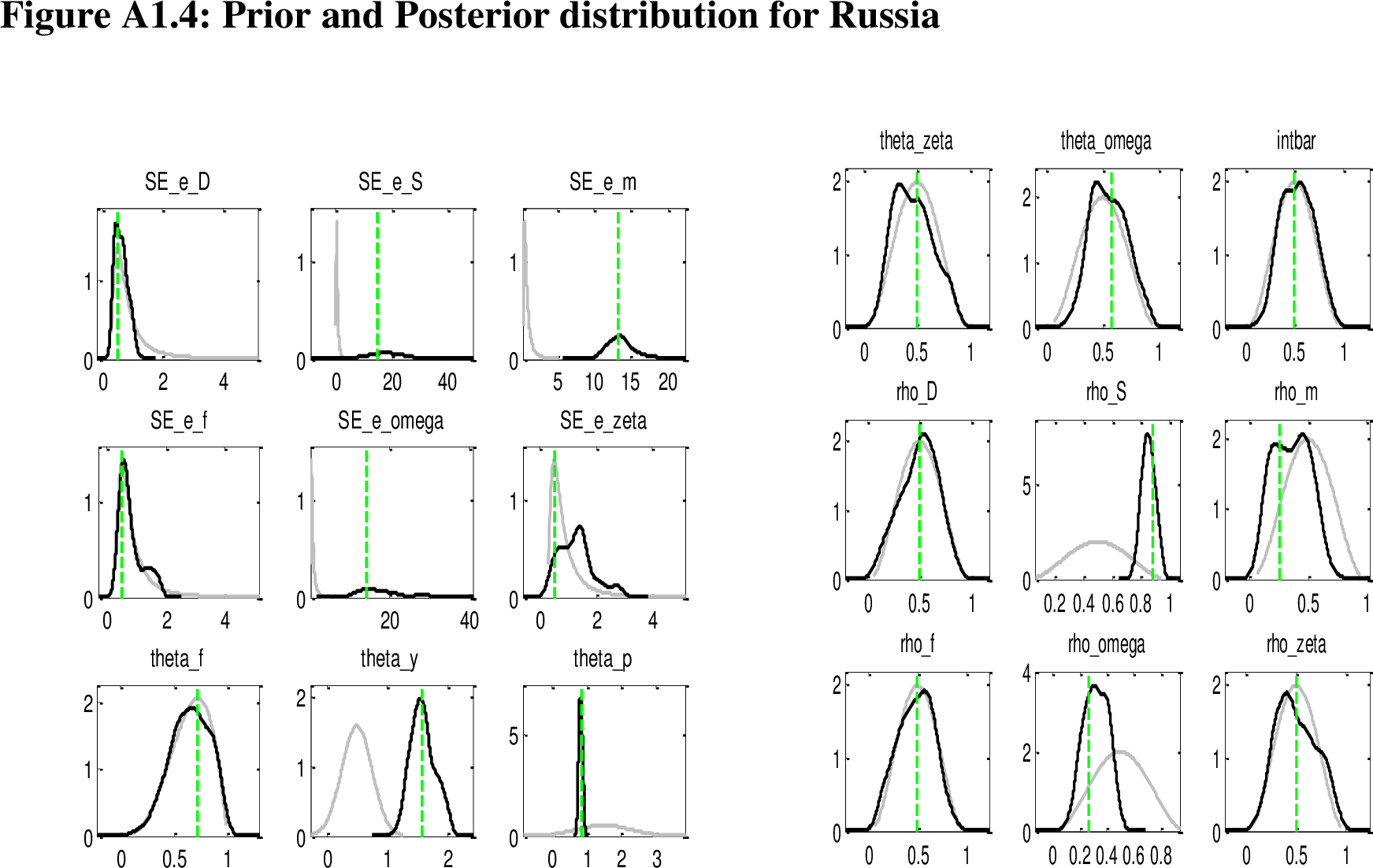

Supplement: S1 Fig — (ZIP) [file pone.0307436.s001.zip › Fig A1.4.tif]

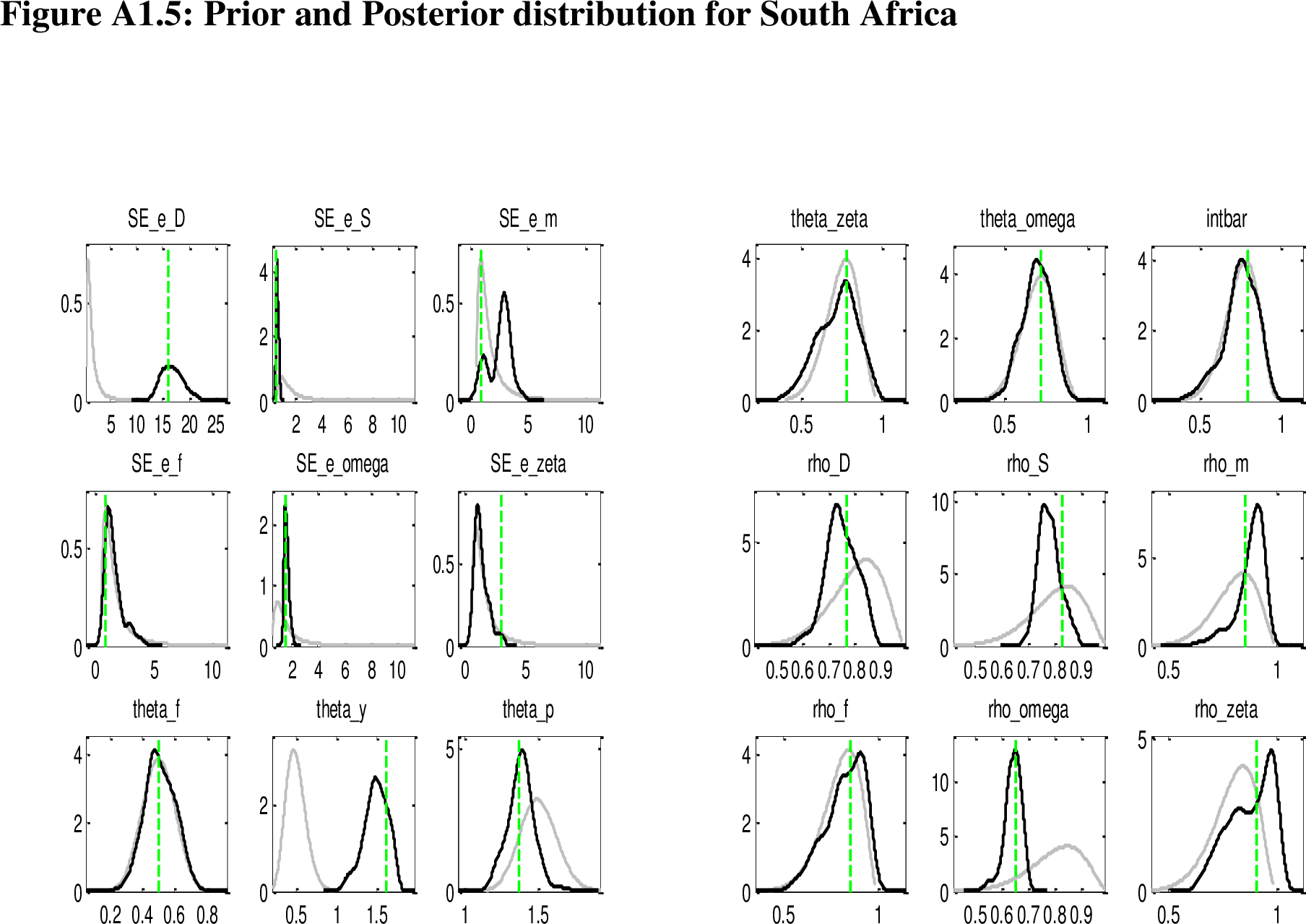

Supplement: S1 Fig — (ZIP) [file pone.0307436.s001.zip › Fig A1.5.tif]

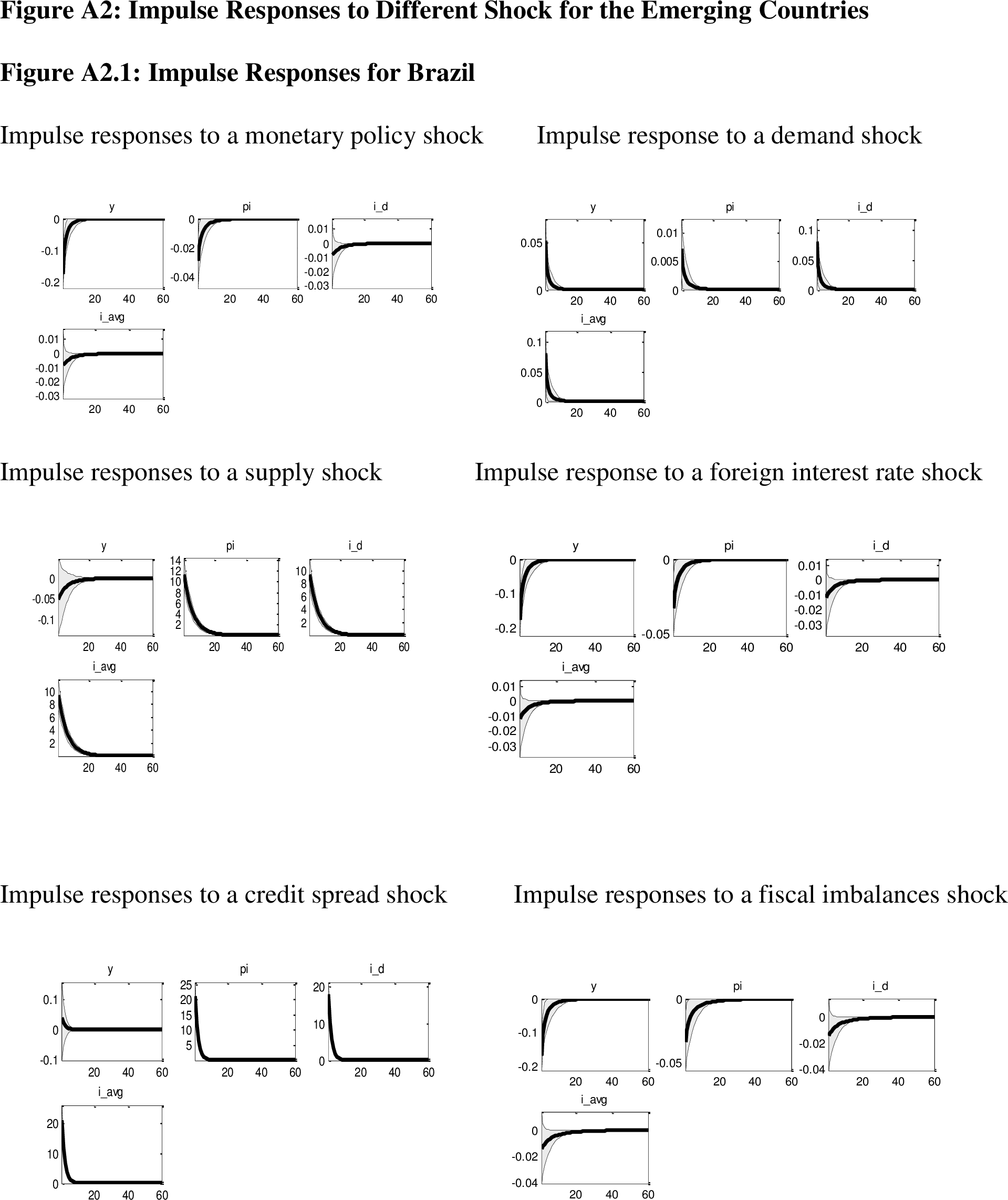

Supplement: S2 Fig — (ZIP) [file pone.0307436.s002.zip › Fig A2.1.tif]

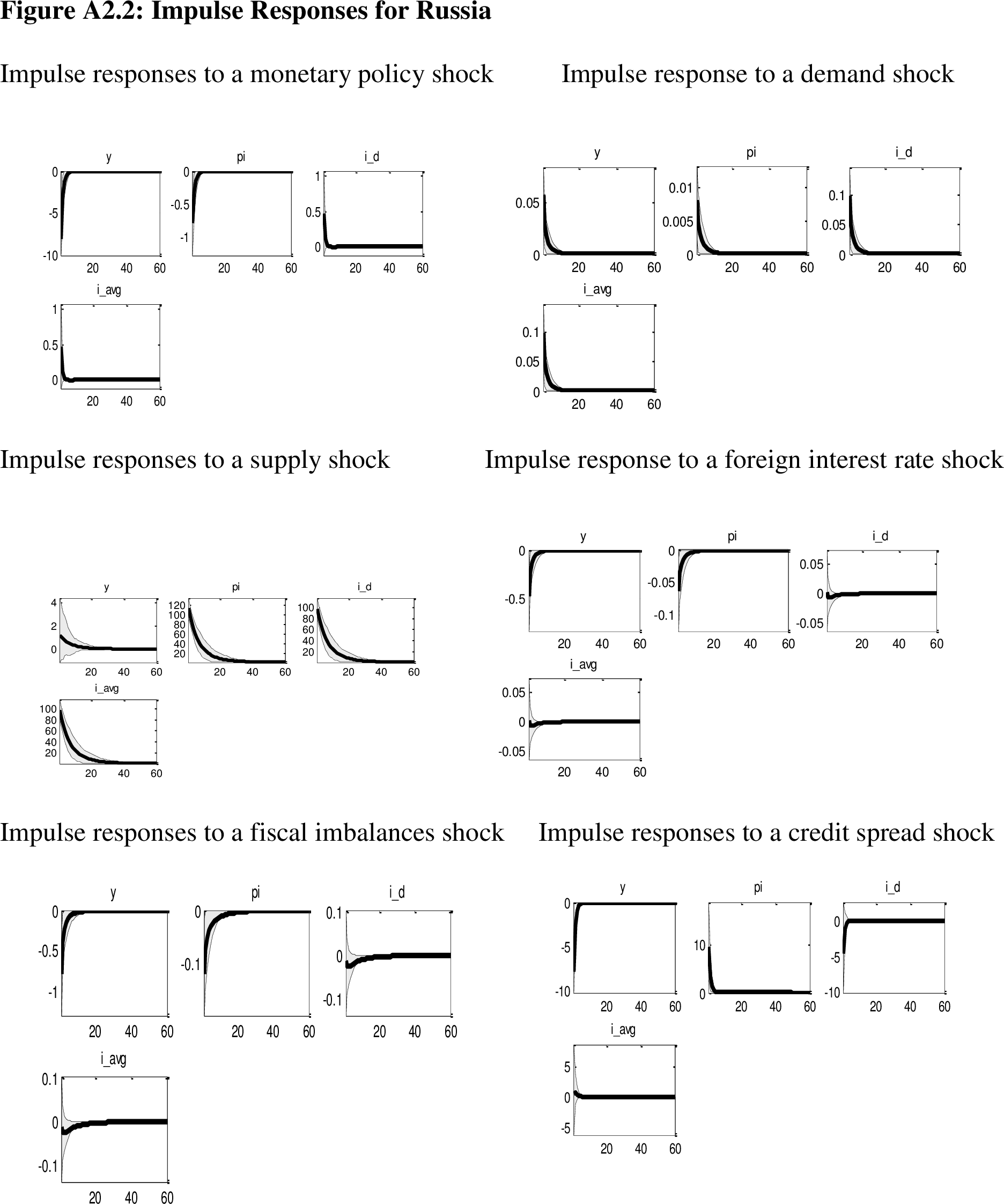

Supplement: S2 Fig — (ZIP) [file pone.0307436.s002.zip › Fig A2.2.tif]

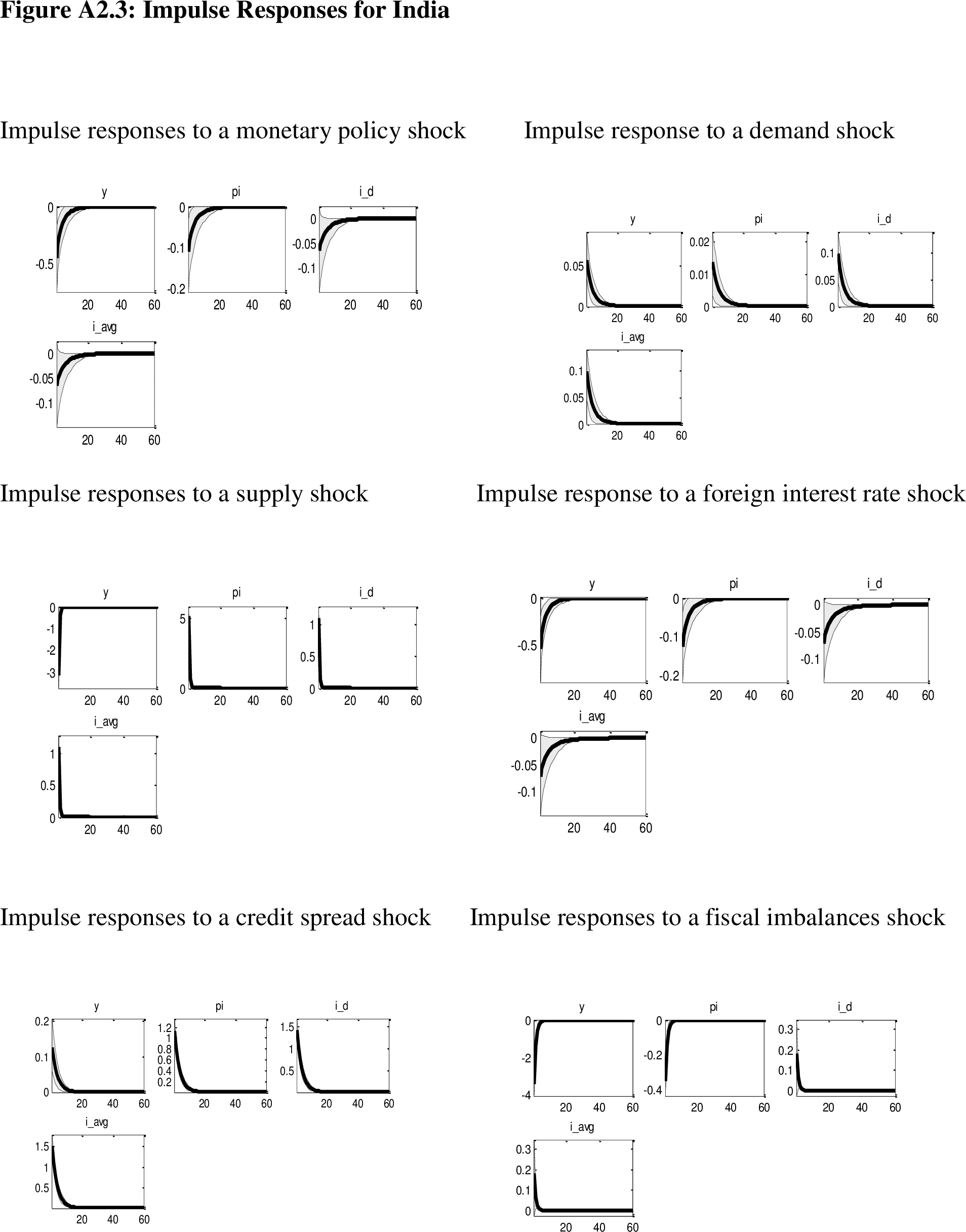

Supplement: S2 Fig — (ZIP) [file pone.0307436.s002.zip › Fig A2.3.tif]

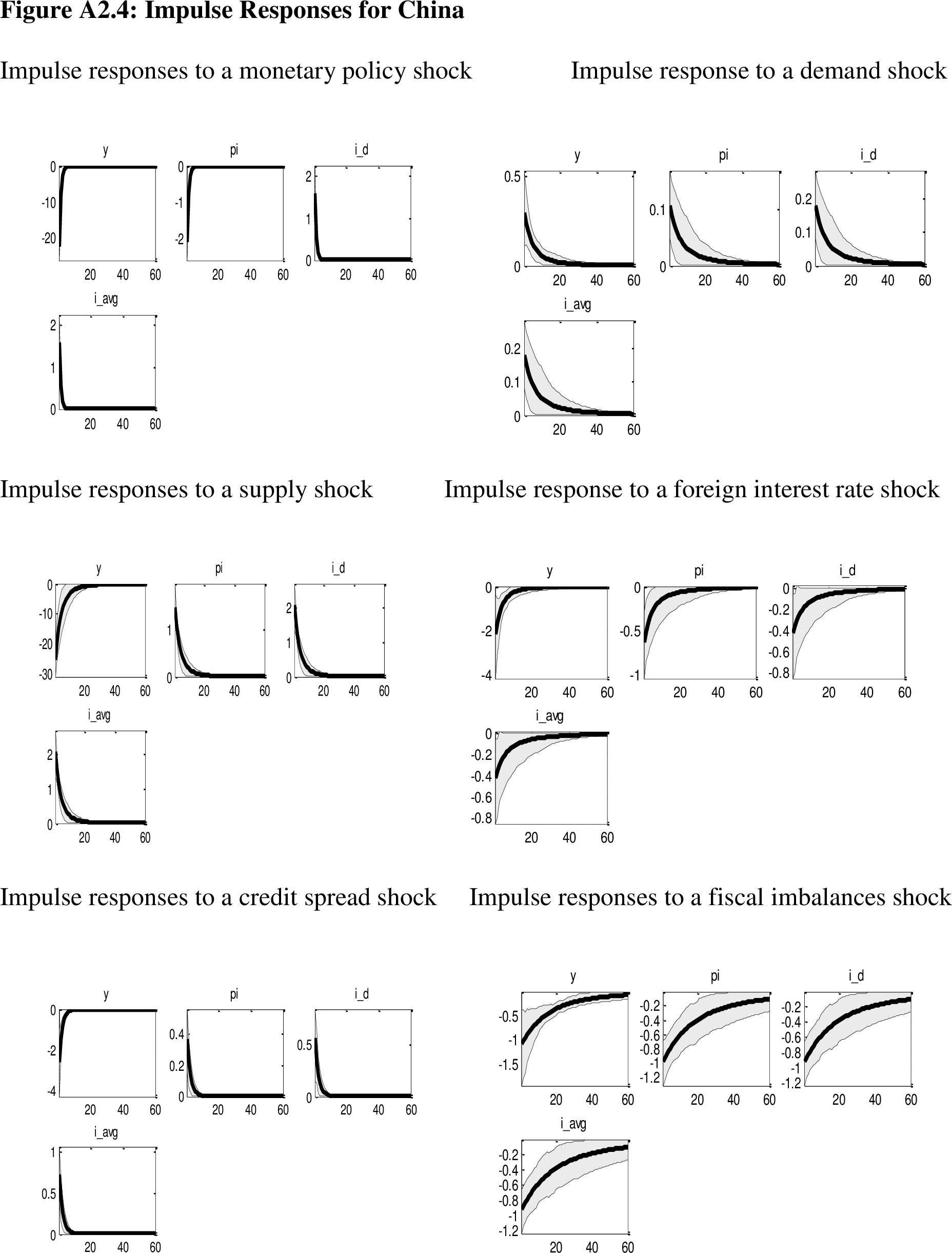

Supplement: S2 Fig — (ZIP) [file pone.0307436.s002.zip › Fig A2.4.tif]

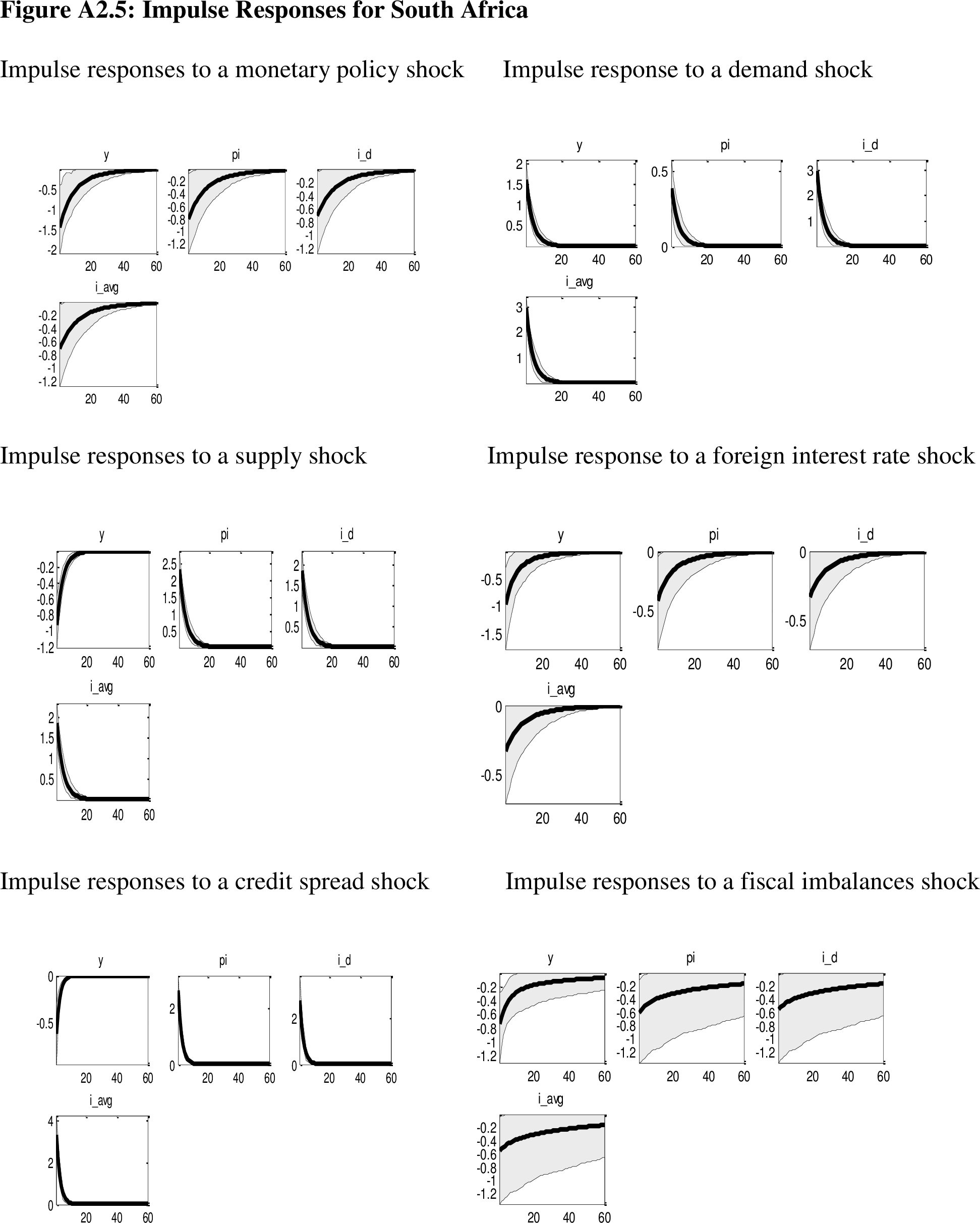

Supplement: S2 Fig — (ZIP) [file pone.0307436.s002.zip › Fig A2.5.tif]
