# Supplementary material for: Monetary policy reaction function: A Bayesian analysis for the BRICS
Source: PLoS One. 2024 Aug 28;19(8):e0307436. doi: 10.1371/journal.pone.0307436 (PMC11355566; doi:10.1371/journal.pone.0307436)
Supplement: S1 Appendix — (DOCX) [file pone.0307436.s003.docx]

**Supporting Information 1**

**S.1 Derivation of Monetary Policy Reaction Function**

Our model comprises of three major sectors viz. household, intermediary sector, which is the central bank of the country, and corporate firms. Below we discuss each of these sectors in detail separately in this section.

### **1.1.1 Household**

Following Cúrdia and Woodford (2016), we assume that households are identical. However, due to independent fluctuations in preferences, they would have heterogeneous preferences. Each type of household wants to maximize an expected discounted utility.

$E_{O} \sum_{t=0}^{\infty} \beta^{t}( u^{\tau_{t} \left( i \right)} \left( c_{t} \left( i \right); \varepsilon_{t} \right)- \int_{0}^{1} v^{\tau_{t}} \left( h_{t}\left( j, i \right); \varepsilon_{t} \right)dj )$ (1. 1)

where,

$u^{\tau}\left( C_{t}\left( i \right) ; \varepsilon_{t} \right) \equiv\frac{{( C_{t} \left( i \right))}^{1- \sigma_{\tau}^{-1}} \left( C_{t}^{-\tau} \right)^{\sigma_{\tau}^{-1}}}{1- \sigma_{\tau}^{-1}}$ (1. 2)

Similarly, the disutility of labor will be

$v^{\tau}\left( h_{t}\left( j,i \right); \varepsilon_{t} \right) \equiv\frac{\varphi_{T} ( h_{t} \left( j,i \right)^{1+v} H_{t}^{-v})}{1+V}$ (1.3)

“j” represents specialized labor hired in different firms.

For $\tau=b,s$ indicating the type of household i.e., borrowers and savers.

$c_{t}$ shows Dixit-Stiglitz aggregate of a household’s purchase of goods.^[[1]](#footnote-1)^

The model also assumes that

$u_{c}^{b}\left( c; \varepsilon\right)>u_{c}^{s}\left( c; \varepsilon\right)$ (1.4)

The marginal utility of a borrower is higher than that of a saver. Thus, borrowers are more impatient than savers. Borrowers, therefore, want to increase present consumption by borrowing and savers want to increase future consumption by saving.

The nominal assets and distributions by intermediaries of household “i” at the beginning of period “t” are:

$A_{t} \left( i \right)=\left( B_{\left( t-1 \right)} \left( i \right) \right)^{+}( 1+ i_{t-1}^{d}$)+$\left( B_{\left( t-1 \right)} \left( i \right) \right)^{-}\left( 1+ i_{t-1}^{b} \right)+T_{t} \left( i \right)+ D_{t}^{int}$ (1.5)

However, the household’s nominal assets at the end of period t-1 will be

$\left( B_{\left( t-1 \right)} \left( i \right) \right)^{+} \equiv\max\left( B_{t-1}\left( i \right), 0 \right)$ (1. 6)

$\left( B_{\left( t-1 \right)} \left( i \right) \right)^{-} \equiv\min\left( B_{t-1}\left( i \right), 0 \right)$ (1. 7)

$T_{t} \left( i \right)$ represents the household’s transfer from the insurance agency

The net assets at the end of period will be given as

$B_{t}\left( i \right)=A_{t} \left( i \right)+ \int W_{t} \left( j \right)h_{t}\left( j,i \right) dj+ D_{t}^{f}+ D_{t}^{int}$+$D_{t}^{FA}+T_{g}^{t}-P_{t}C_{t}\left( i \right)$ (1.8)

where $W_{t}$(j) = Nominal wage for type “j” labor, $T_{g}^{t}=$Lump-sum government transfer, $D_{t}^{f}$ = Distributed profits of the firm, $D_{t}^{int}=$ Distributed profits of financial intermediary sector, $D_{t}^{FA}=$Distributed profits of the financial assets, $P_{t}=$Dixit-Stiglitz price index in period “t”. $D_{t}^{FA}$ is an additional term in the above equation as compared to the earlier models.

The post transfer wealth of households, who has right of entry to the insurance agency at the beginning of period “t” will be

$A_{t}\left( i \right)=A_{t}= \int A_{t} \left( h \right) dh$ (1.9)

For households who do not have access to the insurance agency, the wealth will be measured by equation (3. 5) by considering $T_{t} \left( i \right)=0$

$A_{t} \left( i \right)=\left( B_{\left( t-1 \right)} \left( i \right) \right)^{+}\left( 1+ i_{t-1}^{d} \right)+\left( B_{\left( t-1 \right)} \left( i \right) \right)^{-}\left( 1+ i_{t-1}^{b} \right)+D_{t}^{int}$ (1.9a)

If $d_{t}$ represents aggregate deposits at the end of period “t” and “$b_{t}$” represents aggregate real borrowings from intermediaries, then

$P_{t}b_{t}= - \int A_{t}\left( i \right)di$ (1. 10)

$P_{t}\left( b_{t}^{g}+d_{t} \right)= \int A_{t} \left( i \right)di$ (1. 11)

Putting equation (1.11) in equation (1.5), equation (1. 5) will take the following form

$A_{t}=\left( \left( d_{t-1}+ b_{t-1}^{g} \right)\left( 1+i_{t-1}^{d} \right)-b_{t-1}\left( 1+ i_{t-1}^{b} \right) \right)p_{t-1}+D_{t}^{int}$ (1.12)

Following Curdia and Woodford (2016) the supply of government debt will evolve according to budget constraint of government.

$b_{t}^{g}= b_{t-1}^{g}\frac{1+ i_{t-1}^{d}}{\pi_{t}}+G_{t}+\frac{T_{t}^{g}}{p_{t}}-\tau_{t}y_{t}$ (1.13)

Distributed profits of firms are

$D_{t}^{f}=\left( 1-\tau_{t} \right)p_{t}y_{t}- \int w_{t}\left( j \right)h_{t}\left( j \right)dj$ (1. 14)

$D_{t}^{f}=\left( 1-\tau_{t} \right)p_{t}y_{t}-\pi_{b}w_{t}^{b}-\pi_{s}w_{t}^{s}$ (1. 15)

$D_{t}^{f}$ representing net sales taxes minus the aggregate wage bill of the firm

Incorporating equation (1. 14) in equation (1. 8), equation (1.8) will take the following form

$B_{t}\left( i \right)=A_{t}\left( i \right)+ \int w_{t}\left( j \right)h_{t}\left( j \right)dj+\left( 1-\tau_{t} \right)p_{t}y_{t}- \int w_{t}\left( j \right)h_{t}\left( j \right)dj+ D_{t}^{int}+ D_{t}^{FA}+ T_{t}^{g}-p_{t}c_{t}\left( i \right)$ (1. 16)

By doing further simplification

$B_{t}\left( i \right)=A_{t}\left( i \right)+$ $\left( 1-\tau_{t} \right)p_{t}y_{t}+ D_{t}^{int}+ D_{t}^{FA}+ T_{t}^{g}-p_{t}c_{t}\left( i \right)$ (1. 17)

The evolution of two interest rates $i_{t}^{d}$ and$i_{t}^{b}$ are considered as given by household. In equilibrium, these interest rates are linked as

$1+ i_{t}^{b}=( 1+ i_{t}^{d})(1+\omega_{t})$ (1. 18)

Credit spread is determined as

$\omega_{t}= \omega_{t}\left( b_{t} \right)$ (1. 19)

As cost of real loan is $E_{t}\left( b_{t} \right),$ therefore, in the goods market, the market clearing condition will be

$Y_{t}= \int c_{t} \left( i \right) di+G_{t}+E_{t}\left( b_{t} \right)$ (1. 20)

Optimal household decisions for the household when$A_{t}\left( i \right) >0$ , i.e. for savers it will be

$\lambda_{t}\left( i \right)= \beta\left( 1+ i_{t}^{d} \right)E_{t}( \frac{\lambda_{t+1}(i)}{\pi_{t+1}}$) (1.21)

First order condition of optimal intertemporal allocation of household consumption expenditure shows that a household’s marginal utility of real income in period “t” will be

$$\lambda_{t}\left( i \right)= u_{c}\left( c_{t}\left( i \right); \varepsilon_{t} \right)$$

For borrowers, it has to satisfy the following condition

$\lambda_{t} \left( i \right)=\beta\left( 1+i_{t}^{b} \right)E_{t}(\frac{\lambda_{t+1}(i)}{\pi_{t+1}}$) (1.22)

The marginal utility of income for all households can be described by the following two stochastic processes

$\lambda_{t}^{b}\left( i \right)= \beta E_{t}(1+\frac{i_{t}^{b}}{\pi_{t+1}}( \left( \delta+\left( 1-\delta\right)\pi_{b} \right)\lambda_{t+1}^{s}$)) (1.23)

$\lambda_{t}^{s}\left( i \right)= \beta E_{t}(1+\frac{i_{t}^{d}}{\pi_{t+1}}( \left( \left( 1-\delta\right)\pi_{b}\lambda_{t+1}^{b}+(\delta+\left( 1-\delta\right)\pi_{s} \right)\lambda_{t+1}^{s}$)) (1.24)

Households of specific type must choose same consumption in any period, common level of consumption must satisfy

$\lambda_{t}^{b}= u^{b^{'}}(c_{t}^{b})$ (1.24a)

$\lambda_{t}^{s}= u^{s^{'}}(c_{t}^{s})$ (1.24b)

Therefore, the inversion of equation (1.24a), and (1.24b) results in

$c_{t}^{b}= c^{b}( \lambda_{t}^{b}, \varepsilon_{t})$ (1.24c)

$c_{t}^{s}= c^{s}( \lambda_{t}^{s}, \varepsilon_{t})$ (1.24d)

Putting these in equation (4.20), we get

$y_{t}= \pi_{b}c^{b}\left( \lambda_{t}^{b}, \varepsilon_{t} \right)+\pi_{s}c^{s}\left( \lambda_{t}^{s}, \varepsilon_{t} \right)+G_{t}+E_{t}b_{t}$ (1.25)

Both type of households have identical preferences $u^{b}\left( c;\varepsilon\right)= u^{s}\left( c;\varepsilon\right)$ and$v^{b}\left( h;\varepsilon\right)= v^{s}\left( h;\varepsilon\right)$. It is also assumed that the wedge between deposit and lending rate is zero ($\omega_{t}\left( b \right)=0)$. Thus, equation (1.25) simply reduces to

$y_{t}=C_{t}+G_{t}$ (1.26)

This equation shows that current aggregate expenditures depend on the current consumption and current government expenditure.

### **1.1.2 Intermediary Sector**

A bank collects “$d_{t}"$as deposits. Central bank treats both the legitimate and fraudulent borrowers equally. However, for period t+1, it correctly predicts the loan repayments i.e. $p_{t}b_{t}(1+i_{t}^{b})$. Excess funds that a bank received from the depositors but does not lent out will be distributed to the shareholders. Again by following Cúrdia and Woodford (2016), we define

$D_{t}^{int}=d_{t}-b_{t}-\chi_{t}\left( b_{t} \right)-E_{t}\left( b_{t} \right)$ (1. 27)

Deposit $d_{t}$ is considered to satisfy the following condition

$\left( 1+i_{t}^{d} \right) d_{t}=\left( 1+i_{t}^{b} \right)b_{t}$ (1. 28)

By using equation (1.18), it takes the following form

$d_{t}= \frac{\left( 1+ i_{t}^{b} \right) b_{t}}{(1+ i_{t}^{d})}$ (1.29)

$d_{t}=\left( 1+ \omega_{t} \right)b_{t}$ (1.30)

And real distribution by intermediaries equals to

$D_{t}^{int}=\omega_{t}b_{t}- \chi_{t}\left( b_{t} \right)-E_{t}\left( b_{t} \right)$ (1. 31)

Household income must include the household’s earnings from fraud. Hence, we write

$D_{t}^{int}=p_{t}\left( w_{t}b_{t}-E_{t}\left( b_{t} \right) \right)$ (1. 32)

In order to maximize $b_{t},$ we take derivative of equation (1. 31) w.r.t $b_{t}$, and set equal to zero

$\omega_{t}- \chi_{t}^{'}$ ($b_{t})- E_{t}^{'}\left( b_{t} \right)=0$ (1. 33)

Therefore, in the equilibrium, the credit spread is

$\omega_{t}=w_{t}\left( b_{t} \right)= \chi_{t}^{'}\left( b_{t} \right)+ E_{t}^{'} (b_{t})$ (1.34)

## **1.2 The Dynamics of Private Indebtedness**

By incorporating $D_{t}^{int}$ and $D_{t}^{FA}$ in equation (1.8)

$D_{t}^{int}=p_{t}\left( w_{t}b_{t}-E_{t}\left( b_{t} \right) \right)$

$$D_{t}^{FA}=(1-\tau_{t})(1+ i_{t}^{f})$$

$=1+(1-\chi$)$i_{t}^{f}+ \chi i_{t}$ (1. 35)

$\tau_{t}$ represents the tax rate on the foreign bond holdings. These are capital controls in our model.

Thus, equation (1.8) takes the following form

$B_{t}\left( i \right)=A_{t}\left( i \right)+\left( 1-\tau_{t} \right)p_{t}y_{t}+p_{t}\left( w_{t}b_{t}-E_{t}\left( b_{t} \right) \right)+1+\left( 1-\chi\right)i_{t}^{f}+ \chi i_{t}+T_{t}^{g}-p_{t}c_{t}(i)$ (1.36)

Here $\left( 1-\chi\right)i_{t}^{f}$ and $\chi i_{t}$ are the additional terms in our model as compared to earlier models.

$\tau_{t}= \chi( i_{t}^{f}-i_{t}$) (1. 36a)

where $\chi$ shows capital account position. When$\chi=0$, it shows that capital account is open. If $\chi>0 ,$ then capital controls are in place. Central bank imposes capital tax, when there occurs a difference between home and foreign interest rate.

Integrating equation (3. 8) over all period “t”

$b_{t}=\frac{\delta\left( b_{t-1}+\pi_{s}\omega_{t-1}\left( b_{t-1} \right)b_{t-1}+\pi_{b}E_{t-1}\left( b_{t-1} \right) \right)\left( 1+i_{t-1}^{d} \right)}{\pi_{t}}- \pi_{b}E_{t}\left( b_{t} \right)+\pi_{b}\left( \frac{1+\left( 1-\chi\right)i_{t}^{f}+\chi i_{t}+\delta b_{t-1}^{g}\left( 1+i_{t-1}^{d} \right)}{\pi_{t}}-b_{t}^{g} \right)+\pi_{b}\pi_{s}(\left( c_{t}^{b}-c_{t}^{s} \right)-\left( w_{t}^{b}-w_{t}^{s} \right))$ (1. 37)

$\left( c_{t}^{b}-c_{t}^{s} \right)-\left( w_{t}^{b}-w_{t}^{s} \right)$ is the measure of expenditure imbalances.

Let the industry “j” hires both types of households i.e. borrowers and savers then

$v_{h}^{\tau_{t} (i)} \left( h_{t}\left( j;i \right);\varepsilon_{t} \right)= \lambda_{t}\left( i \right)\frac{w_{t}\left( j \right)}{\mu_{t}^{w}}$ (1. 38)

$w_{t} \left( j \right)$ indicates real wage, whereas, $\mu_{t}^{w}$shows wage mark-up.

Like Benigno and Woodford (2005), the iso-elastic functional form will be

$v^{\tau}\left( h;\varepsilon_{t} \right)\equiv\frac{\psi_{\tau}}{1+v}h^{1+v}H^{-V}$ (1. 39)

where$H_{t}$is an exogenous labor supply disturbance process,$v$ shows an inverse of Frisch elasticity of labor supply,$\psi_{b}, \psi_{s} >0$ are the multiplicative coefficients for borrowers and savers, respectively. The coefficient $v \geq0$is assumed to be same for both types of households.

Market clearing condition takes the following form

$\pi_{b}h_{t}^{b}\left( j \right)+\left( 1-\pi_{b} \right)h_{t}^{s}\left( j \right)=h_{t}(j)$

Solving equation (3.38) for competitive labor supply of each type. By aggregating, we get

$h_{t} \left( j \right)=H_{t}{(\frac{\lambda_{t}w_{t}\left( j \right)}{\psi p_{t}})}^{\frac{1}{v}}$ (1. 40)

where,

$\lambda_{t}\equiv{\psi(( \pi_{b} \left( \frac{\lambda_{t}^{b}}{\psi_{b}} \right)^{\frac{1}{v}}+ \pi_{s} \left( \frac{\lambda_{t}^{s}}{\psi_{s}} \right)^{\frac{1}{v}})}^{v}$ (1. 41)

$\psi={( \pi_{b}\psi_{b}^{-\frac{1}{v}}+ \pi_{s}\psi_{s}^{-\frac{1}{v}})}^{-v}$ (1. 42)

These definitions will be normalized so that,$\lambda_{t}^{b}= \lambda_{t}^{s}$, $\lambda_{t}= \lambda_{t}^{b}= \lambda_{t}^{s}$ and $\psi_{b}=\psi_{s}$ and $\psi=\psi_{b}= \psi_{s}$

We can write equation (1. 38) as:

$\frac{W_{t}\left( j \right)}{p_{t}} = \psi{(\frac{h_{t}\left( j \right)}{H_{t}})}^{v}$ (1.43)

Real wage will be,

$w_{t} \left( j \right)= \psi\mu_{t}^{w}\lambda_{t}^{-1}{(\frac{h_{t}\left( j \right)}{H_{t}})}^{v}$ (1. 44)

### **1.2.1 Firms**

We have to determine the distribution of National Income (NI) between labor and capital in order to know about the dynamics of private indebtedness. By following Benigno and Woodford (2005) the iso-elastic production function has the following form

$y_{t}\left( i \right)=Z_{t}h_{t}{(i)}^{1/\varphi}$ (1.45)

$\varphi\geq1 ,$ while $Z_{t}$ is exogenous

Showing that the demand for labor is

$h_{t}\left( i \right)=({\frac{Y_{t}(i)}{z_{t}})}^{\phi}$ (1.45a)

For the industry “j”, labor market equilibrium is defined as

$w_{t}\left( j \right)= \psi\mu_{t}^{w}\lambda_{t}^{-1}(({\frac{Y_{t}(i)}{z_{t}})}^{\phi}{\frac{1}{\bar{H_{t}}})}^{v}$ (1.45b)

Dixit-Stiglitz preferences implying that the utility of household depends on the quantity purchased of certain composite goods. Following Fiore and Tristani (2013), demand for both government as well as households of the differentiated good “i “is given by

$y_{t} \left( i \right)=Y_{t}{(\frac{p_{t}\left( i \right)}{P_{t}})}^{-\theta}$ (1.46)

where $Y_{t}=$ Demand for composite good

$p_{t}\left( i \right)=$ Price of good “i”

θ>1, constant elasticity of substitution (CES) aggregate of the purchases of the individual goods

Profit function of the firm is given as follows

=$\left( 1-\tau\right)pY\left( \frac{p}{P} \right)^{-\theta}-\psi\mu^{W}\tilde{\lambda}^{-1}{((\frac{Y\left( \frac{P}{p} \right)^{-\theta}}{z}\frac{1}{\bar{H}})}^{V}$ P (${\frac{Y_{t}\left( \frac{p_{t}\left( i \right)}{p_{t}} \right)^{-\theta}}{Z})}^{\phi}$

$p$shows price of an individual firm

$P$ shows aggregate price

Like in Woodford (2003), we assume that all firms will reevaluate the prices. Thus, price $p_{t}\left( i \right)$ will each time remain the same for all firms “i” in the “j” industry.

### **3.2.2 Income Distribution**

To find total demand for labor of type “j”, we integrate equation (1. 44) for the firms in each industry “j”

$\int w_{t}\left( j \right)H_{t}\left( j \right)\mathrm{dj}= \int\frac{\psi\mu_{t}^{w}}{\tilde{\lambda_{t}}}\left( \frac{H_{t}\left( j \right)^{v}}{\bar{H}} \right)H_{t}\left( j \right)dj$ (1. 47)

$\boldsymbol{=}\frac{\psi\mu_{t}^{w}}{\tilde{\lambda_{t}}}{\bar{H_{t}}}^{-v}\int{H_{t}(j)}^{1+v}dj$

Using equation (1. 42), and (1.43), we get

$$\int{H_{t}\left( j \right)}^{1+v}dj=\left( \frac{Y_{t}}{z_{t}} \right)^{\phi\left( 1+v \right)}\int\left( \frac{p_{t}\left( j \right)}{p_{t}} \right)^{-\theta\phi\left( 1+v \right)}dj$$

Thus, equation (3. 47) takes the following form

$$\int w_{t}\left( j \right)H_{t}\left( j \right)\mathrm{dj}= \int\frac{\psi\mu_{t}^{w}}{\tilde{\lambda_{t}}}\left( {\bar{H}_{t}}^{-v} \right)({\frac{Y_{t}}{z_{t}})}^{1+\omega}\Delta_{t}$$

where$w_{y}= \phi\left( 1+v \right)-1 \geq0$

$\Delta_{t} \equiv\int\left( \frac{P_{t}\left( i \right)}{P_{t}} \right)^{-\theta\left( 1+\omega_{y} \right)}$(1. 48)

In the Calvo model (1983) of price adjustment, this dispersion measure evolves according to the law of motion.

$\Delta_{t}=h\left( \Delta_{t-1},\pi_{t} \right)$ (1. 49)

$\Delta_{t}$ shows a measure of price dispersion

Law of motion is defined as

$\Delta_{t}=\alpha\Delta_{t-1}{\Pi_{t}}^{\theta\left( 1+\omega_{y} \right)}+(1-\alpha)({1-\alpha\Pi_{t}^{\theta-1})}^{\frac{\theta\left( 1+\omega_{y} \right)}{\theta-1}}$

Wage income differential in equation (1.37) will be derived as

${\omega_{t}}^{b}-{\omega_{t}}^{s}=\frac{\psi{\mu_{t}}^{w}}{\tilde{\lambda_{t}}\bar{H}_{t}^{v}}({\frac{Y_{t}}{Z_{t}})}^{1+\omega_{y}}\Delta_{t}$ (1. 49a)

Using equation (1.47) for the total wage bill, we can solve for the wage income of household of each type i.e. for borrowers and savers. This solution together with the consumption function equations (1. 24c) and (1. 24d) allows the last term in the brackets in equation (1. 37) to be written as a function of the following form

$B\left( Y_{t},\lambda_{t}^{b},\lambda_{t}^{s},\Delta_{t},\varepsilon_{t} \right)$

The law of motion for private indebtedness $b_{t}$ i.e. equation (1.37) can be written as

$b_{t}=\frac{\delta\left( b_{t-1}+\pi_{s}\omega_{t-1}\left( b_{t-1} \right)b_{t-1}+\pi_{b}E_{t-1}\left( b_{t-1} \right) \right)\left( 1+i_{t-1}^{d} \right)}{\pi_{t}}- \pi_{b}E_{t}\left( b_{t} \right)+\pi_{b}\left( \frac{1+\left( 1-\chi\right)i_{t}^{f}+\chi i_{t}+\delta b_{t-1}^{g}\left( 1+i_{t-1}^{d} \right)}{\pi_{t}}-b_{t}^{g} \right)+\pi_{b}\pi_{s}B(Y_{t},\lambda_{t}^{b},\lambda_{t}^{s},\Delta_{t};\tilde{\xi_{t}})$ (1.50)

$\left( 1-\chi\right)i_{t}^{f}$ and $\chi i_{t}$ are the additional terms of our model in the above equation.

This shows that the real private debt is the function of its own past level, financial disturbances, capital control, policy rate, government debt, and a measure of expenditure imbalance.

### **1.2.3 Aggregate Supply and Fiscal Imbalances**

As in Benigno and Woodford (2005), labor supply model indicates that the equilibrium real marginal cost of supplying output in any industry “j” is equal to

$S_{t}\left( j \right)=\frac{\phi\Psi\mu_{t}^{w}y_{t}{(j)}^{wy}}{Z_{t}^{1+wy}H_{t}^{v}\lambda_{t}}$ (1.51)

By following Calvo (1983), inflation equation takes the following form

$\pi_{t}= \pi\left( Z_{t} \right)$ (1.52)

$Z_{t}$ is a vector of two forward looking variables consist of output, fiscal imbalance, marginal utilities of both borrowers and savers and exogenous disturbances which is the sales tax i.e. $\tau_{t}$

$Z_{t}=G\left( Y_{t},\zeta_{t},\lambda_{t}^{b}, \lambda_{t}^{s}; \varepsilon_{t} \right)+E_{t}\left( g\left( \pi_{t+1}, z_{t+1} \right) \right)$ (1. 53)

where $\zeta_{t}$ = fiscal imbalances, $\lambda_{t}^{b}$= marginal utility of borrowers, and $\lambda_{t}^{s}=$ marginal utility of savers. Here again the fiscal imbalance is an additional term in our model.

## **1.3 Credit Frictions**

Like Cúrdia and Woodford (2016), log linearization of equation (1.23) and equation (1.24) yields

$\lambda_{t}^{b}= i_{t}^{b}-E_{t}\pi_{t+1}+ \chi_{b}E_{t}\lambda_{t+1}^{b}+\left( 1-\chi_{b} \right)E\lambda_{t+1}^{s}$ (1.54)

$\lambda_{t}^{s}= i_{t}^{d}-E_{t}\pi_{t+1}+ \chi_{s}E_{t}\lambda_{t+1}^{s}+\left( 1-\chi_{s} \right)E_{t}\lambda_{t+1}^{b}$ (1.55)

where $\lambda_{t}^{\tau}\equiv\frac{\log\lambda_{t}^{\tau}}{\lambda^{r}}$ for $\tau=b,s$

${\pi_{t}=\log\pi_{t}\chi}_{\tau}= \beta\left( 1+ r^{\tau} \right)\left( \delta+\left( 1-\delta\right)\pi_{\tau} \right)$

Log linearization of equation (4.18) yield

$i_{t}^{b}= i_{t}^{d}+ \omega_{t}$ (1.56)

where$\omega_{t} \equiv log(\frac{1+\omega_{t}}{1+ѿ}$) (1.57)

In order to obtain $\omega_{t}$ as a function of$b_{t}$, we can log linearize equation (1.19) where $b_{t}\equiv\log\left( \frac{b_{t}}{\bar{b}} \right)$

Subtracting equation (3.55) from equation (3.54), we get

$Ω_{t}= \omega_{t}+(\chi_{b}+\chi_{s}-1)+ E_{t}(\lambda_{t+1}^{b}+ \lambda_{t+1}^{s}$)

$Ω_{t}= \omega_{t}+{\delta E_{t}Ω}_{t+1}$ (1.58)

Here, $Ω_{t}= {\lambda_{t}}^{b}-{\lambda_{t}}^{s}$showing a measure of inefficiency of financial intermediation. The marginal utilities of the two types will be equal if the financial markets are frictionless.

Again by using equation (1.54) and equation (1. 55), we have

$\delta\equiv\chi_{b}+ \chi_{s}-1 <1$ ( 1.59)

As $\delta<1,$ so we can solve equation (1.58) on forward basis and it takes the following form

$Ω_{t}= \sum_{j=0}^{\infty} E_{t} \omega_{+j}\delta^{j}$ (1.60)

Log linearization of equation (1.25) will yield

$y_{t}= \pi_{b}c^{b}\left( \lambda_{t}^{b}, Ɛ_{t} \right)+ \pi_{s}c^{s}(\lambda_{t}^{s}$,$Ɛ_{t})+G_{t}+E_{t}b_{t}$ (1. 61)

As $c^{b}= \bar{C}_{t}^{b}{(\lambda_{t}^{b})}^{-\sigma_{b}}$ and $c^{s}= \bar{C}_{t}^{s}{(\lambda_{t}^{s})}^{-\sigma_{s}}$

At steady-state level, above equation takes the following form

$0= \pi_{b}\bar{C}_{t}^{b}{(\lambda_{t}^{b})}^{-\sigma_{b}}+\left( 1- \pi_{b} \right)\bar{C}_{t}^{s}\left( \lambda_{t}^{s} \right)^{-\sigma_{s}}+\hat{E}_{t}b_{t}^{\eta}+\bar{G}-\bar{Y}$ (1. 62)

Now,

$Y_{t}= \pi_{b}s_{b}\left( c_{t}^{b}-\sigma_{b}\lambda_{t}^{b} \right)+\left( 1-\pi_{b} \right)s_{s}\left( \bar{c}_{t}^{s}- \sigma_{s}\lambda_{t}^{s} \right)+E_{t}+ \eta s_{E}b_{t}+G_{t}$ (1. 63)

$Y_{t}=\log\frac{Y_{t}}{\bar{Y}}$ , $\lambda_{t}= \pi_{b}{\lambda_{t}}^{b}+\pi_{s}{\lambda_{t}}^{s},$ $G_{t}=\frac{G_{t}-\bar{G}}{\bar{Y}}$ , $E_{t}=\frac{E_{t}-\bar{E}}{\bar{Y}}$, and$\bar{\sigma}= \pi_{b}s_{b}\sigma_{b}+\pi_{s}s_{s}\sigma_{s} >0$

$Y_{t}=s_{c} \bar{c_{t}}- \bar{\sigma}\left( \lambda_{t}+s_{Ω} Ω_{t} \right)+G_{t}+E_{t}$ (1. 64)

where $s_{c}\bar{c}_{t}= \pi_{b}s_{b}\bar{C}_{t}^{b}+\left( 1-\pi_{b} \right)s_{s}\bar{C}_{t}^{s}$

The variable with a bar represents the steady-state value of that variable. Here $\sigma_{\tau}$ shows elasticity of substitution and it can be shown as $s_{\tau}\equiv\frac{\bar{c_{t}}}{\bar{Y}}$ and $s_{Ω}\equiv\pi_{b}\pi_{s} {(s}_{b}\sigma_{b}-s_{s}\sigma_{s})/\sigmā$

By solving equation (3.64) for $\lambda_{t}$ and putting it in the weighted average of equation (1.54), and equation (1.55),^[[2]](#footnote-2)^ we get

$Y_{t}= -\bar{\sigma} \left( i_{t}^{\mathrm{avg}}-E_{t}\pi_{t+1} \right)+E_{t}Y_{t+1}-s_{c}\bar{c}_{t}-E_{t}\Delta G_{t+1}-E_{t}\Delta E_{t+1}-\bar{\sigma}s_{Ω}Ω+\bar{\sigma}\left( s_{Ω}+\psi_{Ω} \right)E_{t}Ω_{t+1}$

Simplifying the above equation by using equation (1. 52) and (1.53), we get

$Y_{t}= -\bar{\sigma} \left( i_{t}^{avg}-E_{t}\pi_{t+1} \right)+E_{t}Y_{t+1}-E_{t}{\Delta\zeta}_{t+1}-E_{t}\Delta e_{t+1}-\bar{\sigma}s_{Ω}Ω+\bar{\sigma}\left( s_{Ω}+\psi_{Ω} \right)E_{t}Ω_{t+1}$ (1.65)

where,

$i_{t}^{avg}= \pi_{b}i_{t}^{b}+\pi_{s}i_{t}^{d}$ (1.65a)

$\Psi_{Ω}= \pi_{b}\left( 1-\chi_{b} \right)-\pi_{s}\left( 1-\chi_{s} \right)$ (1.65b)

$e_{t}=s_{c}\bar{c}_{t}+E_{t}$ (1.65c)

Equation (1.56) together with equation (1.65a) indicates that the policy rate $i_{t}^{d}$ and IS relation would be associated by the following relation:

$i_{t}^{avg}= \pi_{b}i_{t}^{b}+\pi_{s}i_{t}^{d}$

= $\pi_{b}(i_{t}^{d}+ \omega_{t})+\pi_{s}i_{t}^{d}$

= $\pi_{b}i_{t}^{d}$ + $\pi_{s}i_{t}^{d}$ + $\pi_{b}\omega_{t}$

= ($\pi_{b}$ + $\pi_{s})i_{t}^{d}$+ $\pi_{b}\omega_{t}$

$i_{t}^{avg}=i_{t}^{d}+ \pi_{b}\omega_{t}$ (1.65d)

Similarly, log linearization of aggregate supply block determines the inflation rate. Equation (1.52) and equation (1.53) yield aggregate supply relation. By further simplification of the variables

$\pi_{t}= \xi\left( \omega_{y}Y_{t}- \tilde{\lambda}_{t}-v\bar{h_{t}}-\left( 1+\omega_{y} \right)Z_{t}+ {\mu_{t}}^{w}+\tau_{t} \right)+\beta E_{t}\pi_{t+1}$ (1.66)

where $\bar{\tilde{\lambda}} \equiv\log\left( \frac{\tilde{\lambda_{t}}}{\bar{\tilde{\lambda}}} \right)$ , $\bar{h_{t}}\equiv\log\left( \frac{\bar{H_{t}}}{\bar{H}} \right)$, $Z_{t}\equiv\log\left( \frac{Z_{t}}{\bar{Z}} \right)$ , ${\mu_{t}}^{w}\equiv\log\left( \frac{\mu_{t}^{w}}{\bar{\mu}^{w}} \right)$,

$\tau_{t} \equiv-\log\frac{1-\tau_{t}}{1-\bar{\tau}}$, and $\xi\equiv\frac{1-\alpha}{\alpha}-\frac{1-\alpha\beta}{1+ \omega_{y}\theta}>0$

Average marginal utility of income $\tilde{\lambda}_{t}$ which is the part of aggregate supply relation is not the same like aggregate demand relation. These are linked as

$\tilde{\lambda}=\lambda_{t}+\left( \gamma_{b}-\pi_{b} \right)Ω_{t}$ (1.66a)

$\gamma_{b}= \pi_{b}{(\frac{\Psi\bar{\lambda}^{b}}{\Psi_{b}\bar{\tilde{\lambda}}})}^{1/v}$ (1.66b)

Putting value of $\tilde{\lambda}$ in equation (1.66), then again using equation (1.64) to substitute for$\lambda$, we obtain an aggregate supply relation

$\pi_{t}=k\left( Y_{t}-{Y_{t}}^{n} \right)+\mu_{t}+\xi\left( s_{Ω}+\pi_{b}-\gamma_{b} \right)Ω_{t}-\xi\bar{\sigma}^{-1}(\zeta_{t}+e_{t})+ \beta E_{t}\pi_{t+1}$ (1.67)

where $k\equiv\xi\left( \omega_{y}+ \bar{\sigma}^{-1} \right)>0$, $e_{t}\equiv s_{c}\bar{c}_{t}+E_{t}$, and $\mu_{t} \equiv\xi(\mu_{t}^{w}+ \tau_{t})$

If the central authority follows the following Taylor rule

$i_{t}^{d}=\bar{i}+\theta_{p}\pi_{t}+\theta_{y}Y_{t}+\theta_{f}i_{t}^{*}+\theta_{n}\zeta_{t}+m_{t}$ (1.68)

Putting the above equation in equation (1.65d) yields

${i_{t}}^{avg}=$ $\bar{i}+\theta_{p}\pi_{t}+\theta_{y}Y_{t}+\theta_{f}i_{t}^{*}+\theta_{n}\zeta_{t}+ϴ_{⍵}\omega_{t}+m_{̑t}$ (1.69)

where$\theta_{f}= \theta_{f}^{c}+\theta_{f}^{o}$ represents closed and open capital account respectively

$\theta_{f}$ is normalized on 0-1 scale, 0 represents completely closed capital account and 1 represents completely open capital account. When capital account is open, we introduced the interaction term i.e. $K_{it}$ showing capital account openness index in the country.

1. Building block of modern macroeconomics, easy to aggregate things over a sector or whole economy which make it useful to move from micro relationships towards macro relationships. [↑](#footnote-ref-1)
2. $\hat{\lambda}_{t}=-\bar{\sigma}^{-1}\left( \hat{Y}_{t}-g_{t}-\hat{E}_{t} \right)\hat{\Omega}_{t}-s_{\Omega}\hat{\Omega}_{t}$. [↑](#footnote-ref-2)
