# Supplementary material for: Monetary policy reaction function: A Bayesian analysis for the BRICS
Source: PLoS One. 2024 Aug 28;19(8):e0307436. doi: 10.1371/journal.pone.0307436 (PMC11355566; doi:10.1371/journal.pone.0307436)
Supplement: S2 Appendix — (DOCX) [file pone.0307436.s004.docx]

**S2 APPENDIX**

**Figure A2: Impulse Responses to Different Shock for the Emerging Countries**

**Figure A2.1: Impulse Responses for Brazil**

Impulse responses to a monetary policy shock Impulse response to a demand shock

Impulse responses to a supply shock Impulse response to a foreign interest rate shock

Impulse responses to a credit spread shock Impulse responses to a fiscal imbalances shock

**Figure A2.2: Impulse Responses for Russia**

Impulse responses to a monetary policy shock Impulse response to a demand shock

Impulse responses to a supply shock Impulse response to a foreign interest rate shock


Impulse responses to a fiscal imbalances shock Impulse responses to a credit spread shock

**Figure A2.3: Impulse Responses for India**

Impulse responses to a monetary policy shock Impulse response to a demand shock

Impulse responses to a supply shock Impulse response to a foreign interest rate shock

Impulse responses to a credit spread shock Impulse responses to a fiscal imbalances shock

**Figure A2.4: Impulse Responses for China**

Impulse responses to a monetary policy shock Impulse response to a demand shock

Impulse responses to a supply shock Impulse response to a foreign interest rate shock

Impulse responses to a credit spread shock Impulse responses to a fiscal imbalances shock

**Figure A2.5: Impulse Responses for South Africa**

Impulse responses to a monetary policy shock Impulse response to a demand shock

Impulse responses to a supply shock Impulse response to a foreign interest rate shock

Impulse responses to a credit spread shock Impulse responses to a fiscal imbalances shock
