# Supplementary material for: Monetary policy reaction function: A Bayesian analysis for the BRICS
Source: PLoS One. 2024 Aug 28;19(8):e0307436. doi: 10.1371/journal.pone.0307436 (PMC11355566; doi:10.1371/journal.pone.0307436)
Supplement: Attachment — Submitted filename: 2023.11.pdf [file pone.0307436.s006.pdf]

Referee report

Title: "Monetary Policy Reaction Function: A Bayesian Analysis"

Journal: PLOS One

Manuscript: PONE-D-23-06648R1

This paper estimates monetary policy reaction functions in a DSGE model framework using Bayesian techniques for BRICS and Pakistan from 1970-2021. The paper constitutes an original theoretical integration and empirical estimation of credit spreads and fiscal imbalances in a DSGE framework.

I enjoy this field and the research questions developed in this paper. I will try to provide some insights to improve this paper, hoping they will be helpful in a revision for this or another journal.

Main comments

1. Paper Structure

   The paper is difficult to read and does not expose a clear argumentative path to the research question, which is to estimate MPRFs for specific countries. To address this issue, the authors should clarify the set of countries analyzed and the reason behind this choice. For instance, why include Pakistan? Analyzing and comparing the BRICS MPRFs is already a significant challenge. I suggest the authors focus on the BRICS and compare their results with Pakistan (and maybe other countries that recently entered the BRICS in another section). Alternatively, focus only on BRICS.

2. Methodological Issues

   a) The calibration of priors is not appropriate for a comparative study. All the parameters should be calibrated to the same levels for all countries. Same for density functions. Calibrate the estimations for each country the same to let the data speak.

   b) The paper briefly mentions identification issues. This aspect requires more attention; some tests should be done, and a thorough discussion should appear, particularly regarding the potential sensitivity of results to the fixed parameters. Identification tests of Iskrev (2010), Komunjer and Ng (2011), Qu and Tkachenko (2012),

and Ivashchenko and Mutschler (2020), which all are integrated into the Dynare toolbox, must be calculated, and results displayed or at least presented in the discussion about identification.

3. Policy Research Question

The apparent policy research question is not strong enough to be published. Indeed, running estimations and getting parameters of monetary policy rules is not enough for academic or policy research. The authors should add two critical sections to this paper.

   a) Compare the obtained rules with the historical central bank rate decisions or market interest rates to assess the estimates concerning the MPRF and how close they are to effective rates (reality). This comparison should also include one or two standard rules appropriate to BRICS, and computing the RMSE for all of these rules should demonstrate that the authors' results overperform the classical ones for BRICS in some ways.
   b) The authors should cite and follow the methodology of Benchimol and Fourçans (2019) and at least compute some loss functions to show that their MPRFs are appropriate according to the respective central bank objectives. They could also quickly discuss optimal policy and Benchimol and Bounader (2023).

4. Discussion

Some discussion on critical points should appear in the paper. First, the fact that the considered economies are open or small open, thus being influenced by the foreign economy potentially in a nonlinear way. As transforming the current model into a fully nonlinear model could be a cumbersome task, I suggest the authors discuss Benchimol and Ivashchenko (2021) in their results and policy discussions to provide the reader with a perspective on the nonlinearities that may occur between the domestic and foreign countries. Second, and more generally, a discussion on policy implications derived from these analyses and results is also recommended. Third, the authors should discuss the interaction of the estimated monetary policy functions with uncertainty regarding the

financial market by citing and relating Benchimol, Saadon and Segev (2023) to their results.

Minor comments

5. References
   The below reference appears in the bibliography:
   Korinek, A., & Sandri, D. (2016). Capital controls or macroprudential regulation? Journal of International Economics, 99(S), S27-S42.
   However, this reference is not cited in the text. I suggest the authors check if each reference entry is cited in the text and if each citation appears in the bibliography.

6. Title
   The current title is too general to attract relevant readers. I suggest the authors include "for the BRICS" in their new title.

7. Conclusion
   The concluding remarks should provide clear avenues for future research, building on the insights gained from the current paper.

I hope these comments will improve your paper.

References

Benchimol, J., Fourçans, A., 2019. "Central bank losses and monetary policy rules: A DSGE investigation," International Review of Economics & Finance, Elsevier, vol. 61(C), pages 289-303. 10.1016/j.iref.2019.01.010

Benchimol, J., and Ivashchenko, S., 2021. "Switching volatility in a nonlinear open economy," Journal of International Money and Finance, vol. 110(C), #102287. 10.1016/j.jimonfin.2020.102287

Benchimol, J., and Bounader, L., 2023. "Optimal monetary policy under bounded rationality," Journal of Financial Stability, vol. 67(C). 10.1016/j.jfs.2023.101151

Benchimol, J., Saadon, Y., and Segev, N., 2023. "Stock market reactions to monetary policy surprises under uncertainty," International Review of Financial Analysis, vol. 89(C), #102783. 10.1016/j.irfa.2023.102783

Iskrev, N., 2010. Local identification in DSGE models. Journal of Monetary Economics 57(2), 189-202. 10.1016/j.jmoneco.2009.12.007

Ivashchenko, S., Mutschler, W., 2020. The effect of observables, functional specifications, model features and shocks on identification in linearized DSGE models. Economic Modelling 88(C), 280-292. 10.1016/j.econmod.2019.09.039

Komunjer, I., Ng, S., 2011. Dynamic identification of dynamic stochastic general equilibrium models. Econometrica 79(6), 1995-2032. 10.3982/ECTA8916

Qu, Z., Tkachenko, D., 2012. Identification and frequency domain quasi-maximum likelihood estimation of linearized dynamic stochastic general equilibrium models. Quantitative Economics 3(1), 95-132. 10.3982/QE126
