# Supplementary material for: Monetary policy reaction function: A Bayesian analysis for the BRICS
Source: PLoS One. 2024 Aug 28;19(8):e0307436. doi: 10.1371/journal.pone.0307436 (PMC11355566; doi:10.1371/journal.pone.0307436)
Supplement: Attachment — Submitted filename: 2024.02.pdf [file pone.0307436.s008.pdf]

Referee report

Title: “Monetary Policy Reaction Function: A Bayesian Analysis for the BRICS”

Journal: PLOS One
Manuscript: PONE-D-23-06648R2

This paper estimates monetary policy reaction functions in a DSGE model framework using Bayesian techniques for BRICS from 1970-2021. The paper constitutes an interesting theoretical integration and empirical estimation of credit spreads and fiscal imbalances in a DSGE framework.

The authors did not address the previous comments accordingly. This report tries to reexplain those in order to make this paper acceptable. Alternatively, I would recommend to reject this paper.

Main comments

1. Paper Structure
   Comment well addressed.

2. Methodological Issues
   a) The calibration of priors is still not appropriate for a comparative study. All the parameters should be calibrated to the same levels for all countries. Same for density functions. Calibrate the estimations for each country the same to let the data speak.
   Table 2 shows that this comment was ignored. The calibration of priors refers to both the prior mean and prior SD.
   b) The paper briefly mentions identification issues. This aspect requires more attention; some tests should be done, and a thorough discussion should appear, particularly regarding the potential sensitivity of results to the fixed parameters. Identification tests of Iskrev (2010), Komunjer and Ng (2011), Qu and Tkachenko (2012), and Ivashchenko and Mutschler (2020), which all are integrated into the Dynare toolbox, must be calculated, and results displayed or at least presented in the discussion about identification.
   This comment was not addressed. To address this comment, the authors only added this sentence: “An identification problem occurs

if all the structural parameters are estimated by the Bayesian method. Therefore, we have to fix some of the parameters, in order to estimate the remaining structural parameters and exogenous processes.", which is uninformative and unexplained. What kind of identification issues are occurring? Which identification tests fail and which ones succeed? Cite the authors mentioned to point to the corresponding test. What does the authors mean by ***fixing parameters*** exactly?

3. Policy Research Question

   The apparent policy research question is not strong enough to be published. Indeed, running estimations and getting parameters of monetary policy rules is not enough for academic or policy research. The authors should add two critical sections to this paper.

   a) Compare the obtained rules with the historical central bank rate decisions or market interest rates to assess the estimates concerning the MPRF and how close they are to effective rates (reality). This comparison should also include one or two standard rules appropriate to BRICS, and computing the RMSE for all of these rules should demonstrate that the authors' results overperform the classical ones for BRICS in some ways.

      This comment was also ignored. No comparison with the realized interest rate. Their reference to the introduction is imprecise and unaddresses the comment as well.

   b) The authors should cite and follow the methodology of Benchimol and Fourçans (2019) and at least compute some loss functions to show that their MPRFs are appropriate according to the respective central bank objectives. They could also quickly discuss optimal policy and Benchimol and Bounader (2023).

      This comment was also ignored. The authors have to compute several loss functions (at least several weights) and build these loss functions according to the "ideal" central bank objective and/or according to each central bank objective. Also, their citation of Benchimol and Bounader (2023) is inappropriate; the latter do not deal with forecasting questions.

4. Discussion

Some discussion on critical points should appear in the paper. First, the fact that the considered economies are open or small open, thus being influenced by the foreign economy potentially in a nonlinear way. As transforming the current model into a fully nonlinear model could be a cumbersome task, I suggest the authors discuss Benchimol and Ivashchenko (2021) in their results and policy discussions to provide the reader with a perspective on the nonlinearities that may occur between the domestic and foreign countries. Second, and more generally, a discussion on policy implications derived from these analyses and results is also recommended. Third, the authors should discuss the interaction of the estimated monetary policy functions with uncertainty regarding the financial market by citing and relating Benchimol, Saadon and Segev (2023) to their results.

Comments completely ignored.

Minor comments were mostly well addressed.

References

Benchimol, J., Fourçans, A., 2019. "Central bank losses and monetary policy rules: A DSGE investigation," International Review of Economics & Finance, Elsevier, vol. 61(C), pages 289-303. 10.1016/j.iref.2019.01.010

Benchimol, J., and Ivashchenko, S., 2021. "Switching volatility in a nonlinear open economy," Journal of International Money and Finance, vol. 110(C), #102287. 10.1016/j.jimonfin.2020.102287

Benchimol, J., and Bounader, L., 2023. "Optimal monetary policy under bounded rationality," Journal of Financial Stability, vol. 67(C). 10.1016/j.jfs.2023.101151

Benchimol, J., Saadon, Y., and Segev, N., 2023. "Stock market reactions to monetary policy surprises under uncertainty," International Review of Financial Analysis, vol. 89(C), #102783. 10.1016/j.irfa.2023.102783

Iskrev, N., 2010. Local identification in DSGE models. Journal of Monetary Economics 57(2), 189-202. 10.1016/j.jmoneco.2009.12.007

Ivashchenko, S., Mutschler, W., 2020. The effect of observables, functional specifications, model features and shocks on identification in linearized DSGE models. Economic Modelling 88(C), 280-292. 10.1016/j.econmod.2019.09.039

Komunjer, I., Ng, S., 2011. Dynamic identification of dynamic stochastic general equilibrium models. Econometrica 79(6), 1995-2032. 10.3982/ECTA8916

Qu, Z., Tkachenko, D., 2012. Identification and frequency domain quasi-maximum likelihood estimation of linearized dynamic stochastic general equilibrium models. Quantitative Economics 3(1), 95-132. 10.3982/QE126
