# Supplementary material for: Monetary policy reaction function: A Bayesian analysis for the BRICS
Source: PLoS One. 2024 Aug 28;19(8):e0307436. doi: 10.1371/journal.pone.0307436 (PMC11355566; doi:10.1371/journal.pone.0307436)
Supplement: Attachment — Submitted filename: 2024.03.pdf [file pone.0307436.s010.pdf]

Referee report

Title: "Monetary Policy Reaction Function: A Bayesian Analysis for the BRICS"

Journal: PLOS One
Manuscript: PONE-D-23-06648R2

The authors addressed some of my previous comments, but some are still not addressed appropriately. This report tries to reexplain those in order to make this paper acceptable.

Main comments

1. Paper Structure
   Comment well addressed.

2. Methodological Issues
   a) The calibration of priors is now appropriate for a comparative study. All the parameters are calibrated to the same levels for all countries. **Comment well addressed.**

   b) The paper now mentions identification issues and explains that identification tests of Iskrev (2010), Komunjer and Ng (2011), Qu and Tkachenko (2012), and Ivashchenko and Mutschler (2020), display acceptable results in order to confirm identification. I hope the replication files that will be made available to the public will also confirm this. **All in all, comment well addressed.**

3. Policy Research Question
   The apparent policy research question is not strong enough to be published. Indeed, running estimations and getting parameters of monetary policy rules is not enough for academic or policy research. The authors should add two critical sections to this paper.

   a) Compare the obtained rules with the historical central bank rate decisions or market interest rates to assess the estimates concerning

the MPRF and how close they are to effective rates (reality). This comparison should also include one or two standard rules appropriate to BRICS, and computing the RMSE for all of these rules should demonstrate that the authors' results overperform the classical ones for BRICS in some ways.

Again, comment not addressed. No comparison with the realized interest rate (e.g., RMSE) is presented. The reply "the justification of taking the union of BRICS for analysis is highlighted on page 2" is unrelated to my comment.

b) The authors should cite and follow the methodology of Benchimol and Fourçans (2019) and at least compute some loss functions to show that their MPRFs are appropriate according to the respective central bank objectives.

Comment partly not addressed. The authors have to compute several loss functions (at least several weights) and build these loss functions according to the "ideal" central bank objective and/or according to each central bank objective. More policy discussions should be held around these results. If the authors do not want to process these changes (compute and compare loss functions), they have at least to discuss their results (or those from a previous study) in light of Benchimol and Fourçans (2019). Citing Waheed and Rashid (2021) does not address this comment from the final reader's perspective.

4. Discussion

Some discussion on critical points should appear in the paper. First, the fact that the considered economies are open or small open, thus being influenced by the foreign economy potentially in a nonlinear way. As transforming the current model into a fully nonlinear model could be a cumbersome task, I suggest the authors discuss Benchimol and Ivashchenko (2021) in their results and policy discussions to provide the reader with a perspective on the nonlinearities that may occur between the domestic and foreign countries. Second, and more generally, a discussion on policy implications derived from these analyses and results is also recommended. Third, the authors should discuss the interaction of the

estimated monetary policy functions with uncertainty regarding the financial market by citing and relating Benchimol, Saadon and Segev (2023) to their results.

Comments partly not addressed. The idea of these comments is to provide the reader with more perspectives about the results presented to the authors. Adding one or 2 sentences to cite the suggestes references is not the goal. How your results compare to their results? How these papers can explain some limitations of your results? How these paper can confirm your results? More discussions are needed.

Minor comment

The authors have to check if each paper cited in the text is appropriately referenced in the bibliography, and reciprocally. This is currently not the case.

References

Benchimol, J., Fourçans, A., 2019. "Central bank losses and monetary policy rules: A DSGE investigation," International Review of Economics & Finance, Elsevier, vol. 61(C), pages 289-303. 10.1016/j.iref.2019.01.010

Benchimol, J., and Ivashchenko, S., 2021. "Switching volatility in a nonlinear open economy," Journal of International Money and Finance, vol. 110(C), #102287. 10.1016/j.jimonfin.2020.102287

Benchimol, J., and Bounader, L., 2023. "Optimal monetary policy under bounded rationality," Journal of Financial Stability, vol. 67(C). 10.1016/j.jfs.2023.101151

Benchimol, J., Saadon, Y., and Segev, N., 2023. "Stock market reactions to monetary policy surprises under uncertainty," International Review of Financial Analysis, vol. 89(C), #102783. 10.1016/j.irfa.2023.102783

Iskrev, N., 2010. Local identification in DSGE models. Journal of Monetary Economics 57(2), 189-202. 10.1016/j.jmoneco.2009.12.007

Ivashchenko, S., Mutschler, W., 2020. The effect of observables, functional specifications, model features and shocks on identification in linearized DSGE models. Economic Modelling 88(C), 280-292. 10.1016/j.econmod.2019.09.039

Komunjer, I., Ng, S., 2011. Dynamic identification of dynamic stochastic general equilibrium models. Econometrica 79(6), 1995-2032. 10.3982/ECTA8916

Qu, Z., Tkachenko, D., 2012. Identification and frequency domain quasi-maximum likelihood estimation of linearized dynamic stochastic general equilibrium models. Quantitative Economics 3(1), 95-132. 10.3982/QE126
