# Supplementary material for: Monetary policy reaction function: A Bayesian analysis for the BRICS
Source: PLoS One. 2024 Aug 28;19(8):e0307436. doi: 10.1371/journal.pone.0307436 (PMC11355566; doi:10.1371/journal.pone.0307436)
Supplement: Attachment — Submitted filename: 2024.06.pdf [file pone.0307436.s012.pdf]

Referee report

Title: “Monetary Policy Reaction Function: A Bayesian Analysis for the BRICS”

Journal: PLOS One
Manuscript: PONE-D-23-06648R4

The authors addressed some of the previous comments, but a few are still not addressed appropriately. This short report tries to reexplain those comments to make this paper acceptable.

1. On page 12, the authors do mention that the fit of the model to the data will be analyzed (marginal likelihood), but the fit of each estimation is not assessed. A table comparing the log marginal data densities across all estimations is needed.

2. This adds to my comment that still stays unaddressed. The authors have to compare the realized interest rate, i.e., the interest rate recommended by the estimated model, with the realized interest rate (effective). This can be done through simple RMSE, but any other methodology comparing the global fitting of the model AND the specific fitting of the interest rate is necessary to confirm the robustness and relevance of the estimations for the specific research question. For the moment, this has not been done.

3. On a more general perspective, there is not enough discussion of the results and comparisons with the existing literature. This comment stays up to the fifth round (!), so I conclude the authors do not want to invest efforts in this task, while this would bring value to the final reader and the overall readability of the paper and the originality of the obtained results.

4. On page 2, the sentence "In the recent era, some policy rules have gained extensive attention for designing a visible and an effective monetary policy" should cite this fresh paper: 10.1016/j.jmacro.2024.103604 (and the reference has to appear in the bibliography, see below comment)

5. Again, the authors have to check if each paper cited in the text is appropriately referenced in the bibliography, and reciprocally. For instance, Benchimol and Fourçans (2019) does not appear in the bibliography while it is cited in the main text. Thus carefully checking all the references appearing in the text and the bibliography is necessary.
