# Supplementary material for: Monetary policy reaction function: A Bayesian analysis for the BRICS
Source: PLoS One. 2024 Aug 28;19(8):e0307436. doi: 10.1371/journal.pone.0307436 (PMC11355566; doi:10.1371/journal.pone.0307436)
Supplement: Attachment — Submitted filename: Response to Reviewers Feb 24.docx [file pone.0307436.s007.docx]

**Editorial revisions**

| **Sr. No** | **Comment** | **Decision /section** | **Reference** |
| --- | --- | --- | --- |
| **1** | The paper is difficult to read and does not expose a clear argumentative path to the research question, which is to estimate MPRFs for specific countries. To address this issue, the authors should clarify the set of countries analyzed and the reason behind this choice. I suggest the authors focus on the BRICS and compare their results with Pakistan (and maybe other countries that recently entered the BRICS in another section). | **Incorporated in Introduction** | Thank you for useful suggestions.  The paper structure has been improved by incorporating the recommendation of the reviewer.  Relevant sections are highlighted on **page 2** and **page 4.** |
| **2** | a) The calibration of priors is not appropriate for a comparative study. All the parameters should be calibrated to the same levels for all countries. Same for density functions. Calibrate the estimations for each country the same to let the data speak.  b) The paper briefly mentions identification issues. This aspect requires more attention; some tests should be done, and a thorough discussion should appear, particularly regarding the potential sensitivity of results to the fixed parameters. Identification tests which all are integrated into the Dynare toolbox, must be calculated, and results displayed or at least presented in the discussion about identification | **Incorporated** | a) Suggested changes are incorporated on Page 11, 12 and 13 (highlighted).  b) Suggested changes are incorporated on Page 10.  Furthermore, the analysis is conducted in MATLAB using Dynare tool. |
| **3** | a) Compare the obtained rules with the historical central bank rate decisions or market interest rates to assess the estimates concerning the MPRF and how close they are to effective rates (reality). This comparison should also include one or two standard rules appropriate to BRICS, and computing the RMSE for all of these rules should demonstrate that the authors’ results overperform the classical ones for BRICS in some ways.  b) at least compute some loss functions to show that their MPRFs are appropriate according to the respective central bank objectives. | **Incorporated** | a) the justification of taking the union of BRICS for analysis is highlighted on page 2  b) Already done in the deriving the MPRF and duly referred to Waheed and Rashid (2021). Highlighted on page 4. |
| **4** | Some discussion on critical points should appear in the paper. First, the fact that the considered economies are open or small open, thus being influenced by the foreign economy potentially in a nonlinear way. As transforming the current model into a fully nonlinear model could be a cumbersome task, policy discussions to provide the reader with a perspective on the nonlinearities that may occur between the domestic and foreign countries. Second, and more generally, a discussion on policy implications derived from these analyses and results is also recommended. | **Incorporated** | The suggestion is considered and write up in improved in general. |
| **5** | **References**  The below reference appears in the bibliography: Korinek, A., & Sandri, D. (2016). Capital controls or macroprudential regulation? Journal of International Economics, 99(S), S27-S42. However, this reference is not cited in the text. I suggest the authors check if each reference entry is cited in the text and if each citation appears in the bibliography. | **Incorporated** | Mentioned reference is cross checked and removed. All the in text references are bibliography is verified. |
| **6** | **Title**  The current title is too general to attract relevant readers. I suggest the authors include “for the BRICS” in their new title. | **Incorporated** | The title is revised to include “BRICS”, highlighted on page 1. |
| **7** | **Conclusion**  The concluding remarks should provide clear avenues for future research, building on the insights gained from the current paper. | **Incorporated** | The clear avenue for future research is improved, details added and additions highlighted on page 22. |
