# Supplementary material for: Monetary policy reaction function: A Bayesian analysis for the BRICS
Source: PLoS One. 2024 Aug 28;19(8):e0307436. doi: 10.1371/journal.pone.0307436 (PMC11355566; doi:10.1371/journal.pone.0307436)
Supplement: Attachment — Submitted filename: Response to Reviewers march_24.docx [file pone.0307436.s009.docx]

**Editorial revisions**

| **Sr. No** | **Comment** | **Decision /section** | **Reference** |
| --- | --- | --- | --- |
| **1** | The calibration of priors is still not appropriate for a comparative study. All the parameters should be calibrated to the same levels for all countries. Same for density functions. Calibrate the estimations for each country the same to let the data speak. **Table 2 shows that this comment was ignored. The calibration of priors refers to both the prior mean and prior SD.** | **Incorporated** | Suggested changes are incorporated on Page 12, 13, 16 and 17 (highlighted). |
| **2** | The paper briefly mentions identification issues. This aspect requires more attention; some tests should be done, and a thorough discussion should appear, particularly regarding the potential sensitivity of results to the fixed parameters. Identification tests of Iskrev (2010), Komunjer and Ng (2011), Qu and Tkachenko (2012), and Ivashchenko and Mutschler (2020), which all are integrated into the Dynare toolbox, must be calculated, and results displayed or at least presented in the discussion about identification.  **This comment was not addressed. To address this comment, the authors only added this sentence: “An identification problem occurs 2 if all the structural parameters are estimated by the Bayesian method. Therefore, we have to fix some of the parameters, in order to estimate the remaining structural parameters and exogenous processes.”, which is uninformative and unexplained. What kind of identification issues are occurring? Which identification tests fail and which ones succeed? Cite the authors mentioned to point to the corresponding test. What does the authors mean by fixing parameters exactly?** | **Incorporated** | Thank you for useful suggestions.  Suggested changes are incorporated on Page 11 and 12 (highlighted).  Furthermore, the analysis is conducted in MATLAB using Dynare tool as mentioned at page 11.  Mod files of all sample countries are available on request. |
| **3** | Compare the obtained rules with the historical central bank rate decisions or market interest rates to assess the estimates concerning the MPRF and how close they are to effective rates (reality). This comparison should also include one or two standard rules appropriate to BRICS, and computing the RMSE for all of these rules should demonstrate that the authors’ results overperform the classical ones for BRICS in some ways. | **Incorporated** | Suggested changes are incorporated on Page 22 (highlighted). |
| **4** | The authors should cite and follow the methodology of Benchimol and Fourçans (2019) and at least compute some loss functions to show that their MPRFs are appropriate according to the respective central bank objectives. They could also quickly discuss optimal policy and Benchimol and Bounader (2023). **This comment was also ignored. The authors have to compute several loss functions (at least several weights) and build these loss functions according to the “ideal” central bank objective and/or according to each central bank objective. Also, their citation of Benchimol and Bounader (2023) is inappropriate; the latter do not deal with forecasting questions** | **Incorporated** | Already done in the deriving the MPRF and duly referred to Waheed and Rashid (2021). Highlighted on page 4. Complete derivation is Presented in Appendix.  Suggested changes are incorporated on page 5 and 11. |
| **5** | Some discussion on critical points should appear in the paper. First, the fact that the considered economies are open or small open, thus being influenced by the foreign economy potentially in a nonlinear way. As transforming the current model into a fully nonlinear model could be a cumbersome task, I suggest the authors discuss Benchimol and Ivashchenko (2021) in their results and policy discussions to provide the reader with a perspective on the nonlinearities that may occur between the domestic and foreign countries. Second, and more generally, a discussion on policy implications derived from these analyses and results is also recommended. Third, the authors should discuss the interaction of the estimated monetary policy functions with uncertainty regarding the financial market by citing and relating Benchimol, Saadon and Segev (2023) to their results. **Comments completely ignored.** | **Incorporated** | The suggestion is considered and incorporated in this research study at page 3, 6 and 23(highlighted). |
