# Supplementary material for: Monetary policy reaction function: A Bayesian analysis for the BRICS
Source: PLoS One. 2024 Aug 28;19(8):e0307436. doi: 10.1371/journal.pone.0307436 (PMC11355566; doi:10.1371/journal.pone.0307436)
Supplement: Attachment — Submitted filename: Response to Reviewers may 2024.docx [file pone.0307436.s011.docx]

**Editorial revisions**

| **Sr. No** | **Comment** | **Decision /section** | **Reference** |
| --- | --- | --- | --- |
| **1** | Policy Research Question  The apparent policy research question is not strong enough to be published. Indeed, running estimations and getting parameters of monetary policy rules is not enough for academic or policy research. The authors should add two critical sections to this paper.   a) Compare the obtained rules with the historical central bank rate decisions or market interest rates to assess the estimates concerning the MPRF and how close they are to effective rates (reality). This comparison should also include one or two standard rules appropriate to BRICS, and computing the RMSE for all of these rules should demonstrate that the authors’ results overperform the classical ones for BRICS in some ways. b) The authors should cite and follow the methodology of Benchimol and Fourçans (2019) and at least compute some loss functions to show that their MPRFs are appropriate according to the respective central bank objectives. | **Incorporated** | Thank you for the useful suggestions.  Relevant sections are highlighted on **page 23**  Suggested changes are incorporated on Page 2 |
| **2** | Discussion  Some discussion on critical points should appear in the paper. First, the fact that the considered economies are open or small open, thus being influenced by the foreign economy potentially in a nonlinear way. As transforming the current model into a fully nonlinear model could be a cumbersome task, I suggest the authors discuss Benchimol and Ivashchenko (2021) in their results and policy discussions to provide the reader with a perspective on the nonlinearities that may occur between the domestic and foreign countries. Second, and more generally, a discussion on policy implications derived from these analyses and results is also recommended. Third, the authors should discuss the interaction of the estimated monetary policy functions with uncertainty regarding the financial market by citing and relating Benchimol, Saadon and Segev (2023) to their results. | **Incorporated** | Suggested changes are incorporated on Page 6 and 25 (highlighted).  . |
| **3** | The authors have to check if each paper cited in the text is appropriately referenced in the bibliography, and reciprocally. This is currently not the case | **Incorporated** | All the in text references and bibliography is cross checked. |
